# Management Challenges of Extrapulmonary Nontuberculous Mycobacterial Infection: A Single-Center Case Series and Literature Review

**DOI:** 10.3390/pathogens13010012

**Published:** 2023-12-21

**Authors:** Maja Kiselinova, Leslie Naesens, Diana Huis In ’t Veld, Jerina Boelens, Eva Van Braeckel, Yannick Vande Weygaerde, Steven Callens

**Affiliations:** 1 Department of General Internal Medicine, Ghent University Hospital, 9000 Ghent, Belgium; leslie.naesens@uzgent.be (L.N.); steven.callens@uzgent.be (S.C.); 2 Department of Microbiology, Ghent University Hospital, 9000 Ghent, Belgium; jerina.boelens@uzgent.be; 3 Department of Respiratory Medicine, Ghent University Hospital, 9000 Ghent, Belgiumyannick.vandeweygaerde@uzgent.be (Y.V.W.); 4 Department of Internal Medicine and Paediatrics, Faculty of Medicine and Health Sciences, Ghent University, 9000 Ghent, Belgium

**Keywords:** nontuberculous mycobacteria, case series, mycobacterial treatment, mycobacterial resistance, host immune response

## Abstract

Extrapulmonary nontuberculous mycobacterial (NTM) disease remains largely enigmatic, yet these mycobacteria are increasingly acknowledged as important opportunistic pathogens in humans. Traditionally, NTM infections have been identified across various anatomical locations, with the respiratory system being the most affected and best understood. Historically, extrapulmonary NTM infection was predominantly associated with HIV/AIDS, with Mycobacterium avium lymphadenopathy being the most commonly reported. Today, however, because of the expanding utilization of immunosuppressive therapies and the demographic shift towards an aging population, an increasing number of NTM infections are expected and seen. Hence, a heightened index of suspicion is essential, necessitating a multifaceted approach to identification and drug sensitivity testing to improve treatment outcomes. In extrapulmonary NTM management, expert consultation is strongly recommended to determine the most efficacious treatment regimen, as individualized, patient-tailored therapies are often required. Furthermore, the economic burden of NTM disease is considerable, accompanied by high rates of hospitalization. To optimize the management of these intricate infections, there is an urgent need for comprehensive data on incidence, prevalence, and outcomes. This case-based series delves into the intricate nature of extrapulmonary NTM infections, focusing on both rapid and slow-growing NTM species, and explores therapeutic options, resistance mechanisms, and host-related immunological factors.

## 1. Introduction

The epidemiological characteristics of extrapulmonary nontuberculous mycobacterial (NTM) disease remain largely unknown, as there is no systematic reporting or standardized registration of NTM infections in most countries. While NTM are increasingly acknowledged as significant opportunistic pathogens in humans, this recognition is largely for NTM pulmonary disease (NTM-PD), which accounts for about 90% of NTM-related disease [1]. NTM are a heterogenous group of bacteria that consist of approximately 200 species, excluding *Mycobacterium tuberculosis* complex and *Mycobacterium leprae*, and distinguishing between them at the subspecies level is crucial for selecting the most appropriate treatment regimen [2]. A recent genome-based revision of taxonomy has been undertaken with the sequencing of all known species within the genus, with the aim of correct identification of all that can guide diagnosis and treatment [3]. NTM are widely distributed in the environment, as they are natural inhabitants of soil and water and can be cultured from different human samples [3,4]. NTM are acid-fast bacilli that are clinically categorized as rapid growers, usually taking 7–10 days to grow on culture, or slow growers, which may need up to 14 days to grow [5]. Most, with few exceptions, are opportunistic pathogens [4].

Exposure of humans to environmental NTM is almost universal and begins in early childhood. Extrapulmonary NTM infections are mainly seen as cervical lymphadenitis in children. In general, NTM infections are described at different anatomical sites, most commonly and best known in the respiratory system. However, osteoarticular, disseminated, and skin and soft tissue infections have increasingly been reported in the last decade, presumably through iatrogenic or traumatic inoculation [6]. A disseminated extrapulmonary infection attributable to these ubiquitous pathogens indirectly indicates the likelihood of an underlying immune defect, which could be either primary or acquired. In the context of disseminated disease, it is advisable to assess patients for both primary and acquired impairments in cell-mediated immunity. This approach has gained significance owing to the increasing incidence of infections among individuals who appear to be immunocompetent [6,7].

The advancements in diagnostic methodologies have undoubtedly facilitated the enhanced recovery of NTM from human isolates in recent years. The broader availability of molecular diagnostic techniques, such as polymerase chain reaction (PCR) and next-generation sequencing, has allowed for more rapid and accurate identification at the species and subspecies levels. This improvement is not solely attributable to technological progress; it also reflects a heightened level of clinical suspicion among healthcare providers. As diagnostic tools have become more sensitive and specific, clinicians are increasingly able to detect NTM infections that might have previously gone unnoticed or misdiagnosed.

This case series delves into the intricate landscape of complex extrapulmonary NTM infections and aims to elucidate the multifaceted challenges clinicians face in diagnosing and managing these infections.

## 2. Description of Cases

### 2.1. Case 1

A 57-year-old patient with a history of mitral valve annuloplasty (2014) was admitted to the hospital because of increasing back pain and constant thoracic pressure over the left-sided ribcage. There was no report of fever or inflammation in the bloodwork. Magnetic resonance imaging (MRI) confirmed spondylodiscitis at the level of thoracic vertebrae 8 and 9 (T8-T9). Cultures from a biopsy performed in this region were positive for *Cutibacterium acnes*. The patient was empirically treated with ceftriaxone for 6 weeks, but because of progressive back pain and hypoesthesia, a control MRI was performed that showed progression of infection with local abscess formation. Due to clinical signs of progression of spondylodiscitis, new cultures were taken that turned negative, so empirical treatment with moxifloxacin was started. A few weeks later, the patient was readmitted with an ischemic stroke. On the transesophageal echocardiogram, a mitral valve endocarditis was suspected, and the valve was replaced with negative perioperative cultures. No mycobacterial culture was performed. As the infection deteriorated despite treatment, new diagnostic biopsies were taken, and mycobacterial cultures grew *Mycobacterium chimaera*. Because of the medical history of mitral valve surgery in 2014 and the specific culture result, a tentative diagnosis of endocarditis with spondylodiscitis due to *M. chimaera* was made. Empirical treatment with azithromycin, ethambutol, and rifampicin was started. Drug susceptibility testing (DST) showed susceptibility for macrolides, and moxifloxacin was added to the regimen after 8 weeks in view of its penetration at the infection site. Despite 9 months of treatment, progression of spondylodiscitis on MRI was seen, after which surgical debridement was performed and antimicrobial treatment was scaled up with amikacin intravenously (IV) (3 times weekly) and clofazimine. The treatment duration was 22 months in total, with regular MRI follow-up and minimal drug-related toxicity. Until now, >2 years after treatment cessation, there has been no report of relapse.

### 2.2. Case 2

A 70-year-old patient with minimal change nephropathy and nephrotic syndrome, for which he received high doses of corticosteroids in the past, and cardiac failure was admitted to the hospital with increasing back pain over several weeks and tingling sensations in both feet since recently. MRI of the spine revealed spondylodiscitis at levels T9–T10. Surgical debridement was performed, and conventional (non-mycobacterial) peri-operative cultures were negative. The patient was treated empirically with flucloxacillin IV for 2 weeks and subsequently with trimethoprim–sulfamethoxazole in combination with levofloxacin for another 2 weeks, to no avail. A second surgery with laminectomy (levels T8–T9–T10) was required because of progressive neurological damage (paraparesis and hypoesthesia). Empirical antibiotic treatment was changed to vancomycin and high-dose (HD) ceftriaxone (2 grams bid) for 4 weeks. Due to the lack of improvement and persistence of the epidural collection, another surgical drainage was warranted. This time, a mycobacterial culture was performed, growing *Mycobacterium xenopi*. The antibiotic regimen was changed to clarithromycin, ethambutol, rifabutin, and moxifloxacin. Under this combination, an allergic reaction-type drug rash with eosinophilia and systemic symptoms (DRESS) developed, and the antibiotics were stopped. Re-introduction with rifabutin and protionamide was attempted. Linezolid was added to the treatment, with poor tolerance due to the development of bone marrow toxicity in the short term. Because of moderate clinical improvement and poor physical and biological tolerance of the antibiotics, treatment was stopped. Almost 2 years later, relapse of symptoms was reported with fast-evolving paraparesis and epidural collections in T9-T10 on MRI, for which antibiotic treatment was reinitiated with clarithromycin, ethambutol, moxifloxacin, and HD ceftriaxone (2 grams bid). Surgical options were discussed with the patient, but eventually conservative treatment with continuation of antibiotic therapy was opted for. The patient developed new DRESS and antibiotics were put on hold. Another stepwise reintroduction of antibiotics was attempted with rifabutin and amikacin IV. This combination again caused an allergic reaction, resulting in the cessation of all antibiotics. Over the course of several weeks, the patient deteriorated further and died.

### 2.3. Case 3

A 55-year-old patient with a history of kidney transplant and immunosuppressive treatment with tacrolimus, low-dose corticosteroids, and mycophenolate mofetil (MMF) presented to the hospital with an accidental crush trauma of the right index finger after exposure to an aquarium with a persistent wound, cellulitis, and tenosynovitis. Initial empirical treatment with amoxicillin–clavulanic acid for 10 days was not successful. An MRI of the hand revealed osteomyelitis of distal interphalangeal (DIP) II with associated tenosynovitis. Subsequently, surgical debridement was performed, and intraoperative cultures returned positive for *Mycobacterium marinum*, susceptible to macrolides. Combination therapy with azithromycin, ethambutol, and rifabutin (preferred over rifampicin due to interactions with tacrolimus and MMF) was prescribed. However, 2 weeks after treatment initiation, fever, deep neutropenia, and deterioration of liver values developed, presumably toxic effects of the treatment. The treatment was interrupted. After the recovery of both leucopenia and liver enzymes, therapy was restarted with ethambutol and clarithromycin. Rifabutin was omitted to avoid cytopenia. The patient was treated for 6 months. To date, >12 months after treatment cessation, no relapse has been reported.

### 2.4. Case 4

An 82-year-old patient with a medical history of bilateral gonarthrosis and coxarthrosis and a total hip replacement on the left side consulted the emergency department because of a persistent painful wound in the middle of the right foot after an intraarticular infiltration with corticosteroids 4 weeks prior. Shortly after the injection, redness, accompanied by swelling and pain, occurred without clinical improvement after 1 week of oral amoxicillin–clavulanic acid. At presentation, the vital signs were reassuring; no fever was reported, and there were no systemic symptoms. A purulent erysipelas was diagnosed, prompting empirical therapy with IV flucloxacillin and ceftazidime with an unfavorable evolution, necessitating a debridement that revealed a deep purulent infection. Perioperatively obtained deep bone and tissue cultures grew *M. abscessus* subspecies *massiliense*. The targeted therapy consisted of tigecycline, azithromycin, clofazimine, and amikacin. After 2 weeks of treatment, a second surgical debridement was necessary due to an unfavorable evolution. Six weeks post-surgery, amikacin was stopped because of nephrotoxicity, and imipenem was associated. After 3 months of treatment, tigecycline was discontinued because of gastrointestinal intolerance and weight loss. As there was a favorable clinical evolution, 6 months after surgical debridement, the regimen was changed to an all-oral regimen with azithromycin, clofazimine, and doxycycline, ruling out trimethoprim–sulfamethoxazole and linezolid as options due to the potential toxicities. The total duration of treatment was one year. Currently, there is no sign of a relapsing infection (>5 months after treatment cessation).

### 2.5. Case 5

A 78-year-old patient with rheumatoid factor-negative polyarthritis and systemic sclerosis received intra-articular corticosteroid injections in the left foot. Shortly thereafter, amoxicillin–clavulanic acid was given because of a clinical presentation of cellulitis around the left medial malleolus. Four weeks after presentation, an MRI of the foot showed avascular necrosis of the talus with surrounding tibiofibular osteomyelitis and diffuse oedema of the soft tissues. The wound persisted and evolved into an ulcer, for which the patient continued taking different empirical antibiotic treatments (amoxicillin–clavulanic acid, trimethoprim–sulfamethoxazole, and levofloxacin). Initial soft tissue cultures grew *Corynebacterium* spp. Because of a lack of resolution, almost 2 months after the first presentation, a new surgical debridement was performed with perioperative cultures growing *Mycobacterium chelonae*. Initially, the mycobacterium was not regarded as causative for the infection, and the patient was treated empirically with levofloxacin and clindamycin up to 4 months after initial presentation, after which the treatment was changed to azithromycin, moxifloxacin, and IV meropenem for 6 weeks. Because of a favorable evolution, the meropenem was stopped, and dual oral treatment was continued. The patient was treated for a total of 7 months. Six months after the cessation of antibiotic treatment, a relapse occurred, and a new surgical debridement was undertaken. Perioperative cultures were negative. Because of the clinical presentation at that time and the unfavorable evolution under treatment, a lower limb amputation was performed. The patient is not in follow-up anymore.

### 2.6. Case 6

A 32-year-old patient, with no significant medical history, had a cruciate ligament repair at the left knee. Two weeks after surgery, an active fluid build-up at the surgical site was observed, lacking any other signs of complications. A short course of antibiotics was given with no clinical improvement. In the subsequent 4 weeks, his knee function deteriorated further, so drainage was performed. Empirical broad-spectrum antibiotics included vancomycin and ceftazidime, complicated by acute kidney injury, for which the antibiotics were adjusted to ceftriaxone and subsequently trimethoprim–sulfamethoxazole. Peroperative cultures grew *Staphylococcus capitis,* and mycobacterial cultures were eventually positive for *Mycobacterium fortuitum*. A few days after diagnosis, the patient underwent another surgical debridement, and the foreign material was removed. The patient was started on a triple antibiotic regimen with IV tigecycline, amikacin, and HD levofloxacin for 6 weeks, followed by an oral regimen with levofloxacin, doxycycline, and trimethoprim–sulfamethoxazole. The patient stopped treatment after 6 months with no signs of infection up to date (>8 months) (Table 1).

## 3. Diagnosis of Extrapulmonary NTM Infection

The clinical presentation of NTM infection varies considerably, influenced by factors such as the site of infection, host characteristics, the extent of the infection, and the specific bacterial species involved. Disseminated NTM infections may manifest through nonspecific constitutional symptoms such as fever, unexpected weight loss, and nocturnal perspiration. For instance, osteoarticular NTM infections may exhibit indicative signs and symptoms such as joint discomfort, rigidity, erythema, edema, muscle atrophy, and the presence of draining sinuses. Cutaneous NTM infections, on the other hand, may display localized symptoms, including erythema, edema, and pain [6].

The diagnosis of NTM infections remains a complex endeavor, compounded by the absence of universally accepted diagnostic criteria. Several challenges contribute to this complexity. First, clinicians often do not consider a mycobacterial etiology until a patient has failed to respond to broad-spectrum antibiotics, necessitating a higher degree of clinical suspicion for an accurate diagnosis. Second, standard microbiological culture media are not conducive to mycobacterial growth. Specific culture media must be requested, and in some cases, particular incubation conditions are required (e.g., *M. marinum* ideally requires an incubation temperature of 30 °C). Identification techniques generally rely on commercially available molecular systems, such as PCR, which can identify clinically relevant mycobacteria either directly from samples or from cultured isolates. However, these tests come with limitations: they are primarily available in well-equipped reference laboratories, are technically demanding and costly, and may exhibit low accuracy in certain instances (for example, due to inadequate PCR amplification). Third, another complicating factor is the potential inaccessibility of the infection site for obtaining a representative sample, further complicating the diagnostic process.

The current identification of NTM is based on molecular tests for species and subspecies. These tests include PCR analysis, gene probes, and line probe assays (LPA) [8,9,10]. These molecular tests identify a limited number of NTM species but fail to differentiate genetically closely related NTM species. Limitations of conventionally used 16S rRNA gene sequencing include the inability to distinguish subspecies (e.g., *M. abscessus* complex) as well as misidentification (e.g., *M. chimaera* as *M. intracellulare)*. Consequently, DNA sequencing has emerged as the most widely accepted and utilized method for further identification at the subspecies level, with whole genome sequencing (WGS) being considered the gold standard for NTM species identification, providing additional information on mycobacterial characteristics such as virulence and resistance to various antimicrobial agents [10]. Nonetheless, DNA sequencing is a costly technique that necessitates specialized expertise and is not commonly available in standard laboratory settings for NTM diagnosis.

## 4. Treatment of Extrapulmonary NTM Infection

Treatment of extrapulmonary NTM infection varies depending on the species and, in some cases, subspecies, the extent of disease, underlying comorbidities, and drug susceptibility testing, with the most important limitation being that in vitro DST does not predict the clinical response [8,9]. Regimens require the use of multiple antibiotics that are often associated with clinically important adverse reactions and must be administered for prolonged periods.

The treatment is multifaceted. It consists of a combination of antimicrobial therapy and potential source control surgery for an optimal outcome. Adjuvant surgical intervention is described as an option for selected patients with failed medical management, drug-resistant isolates, drug toxicity, or infection-related complications [8]. However, this depends on the extension of the disease and the surgical options. For example, the treatment of uncomplicated NTM lymphadenitis involves complete surgical resection of the involved lymph nodes, and this has been shown to be superior to antibiotic treatment in a randomized control trial [11].

## 5. Cornerstones of Antibiotic Treatment

Experts recommend employing a combination antimicrobial regimen for the treatment of complex NTM infections. While there are currently no established guidelines specifically for disseminated NTM disease, clinicians can refer to the joint ATS/ERS/ESCMID/IDSA guidelines and the consensus management recommendations for less common NTM isolates, both focusing on NTM-PD, to guide their antimicrobial regimen [8,9]. In the best interest of the patient, it is essential that the therapeutic regimen for NTM infections be formulated in consultation with an infectious disease specialist or microbiologist with expertise in managing these conditions. The disseminated nature of the infection usually mandates the initiation of intravenous therapy during the early weeks, commonly known as the induction phase. This is typically followed by a more prolonged treatment course lasting several weeks or months, termed the continuation phase.

In clinical practice, the management of NTM infections frequently becomes more complex due to the need for alterations or adaptations in the drug regimen. This complexity can arise from various factors, such as drug–drug interactions, toxic effects, intolerance, side effects, or allergic reactions. Optimal management of complex NTM infections calls for a multidisciplinary approach, commencing at the onset of treatment and extending through patient follow-up. This integrated strategy necessitates the involvement of specialists across various disciplines, including infectious disease experts, microbiologists, pharmacists, pneumologists, surgeons when needed, and radiologists.

**Figure 1:**
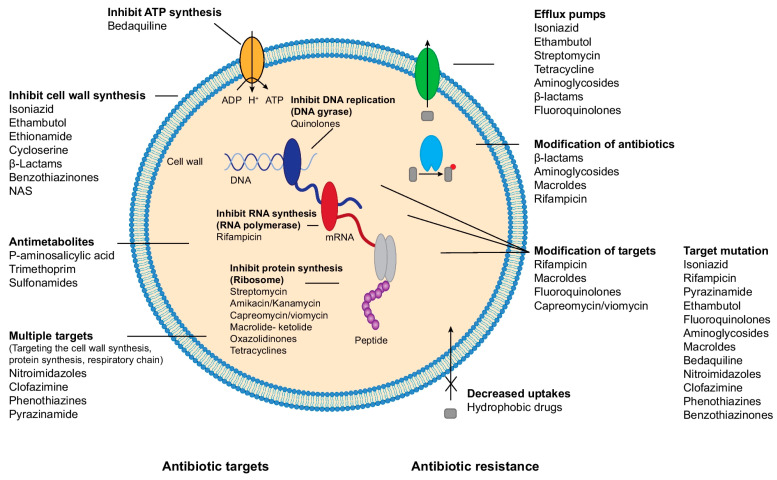
The antibiotic treatment and resistance mechanisms in NTM infections. * Re-printed figure Full text: Nasiri MJ, Haeili M, Ghazi M, Goudarzi H, Pormohammad A, Imani Fooladi AA, et al. New insights into the intrinsic and acquired drug resistance mechanisms in mycobacteria. *Front. Microbiol.* **2017**, *8*, 681. (https://pubmed.ncbi.nlm.nih.gov/28487675/, (accessed on 19 December 2023)).

There are several classes of antibiotics used in the treatment of NTM infections, albeit a limited number of available active drugs (Table 2). The largest group are the ribosome-targeting antibiotics that bind to different sites or different subunits of the ribosome and subsequently block protein synthesis by different mechanisms (Figure 1) such as macrolides, aminoglycosides, tigecycline, and tetracyclines. Rifampicin, an ansamycin antibiotic, blocks DNA-templated RNA synthesis, while quinolones and sulfamethoxazole will suppress DNA synthesis. A fourth group of antibiotics suppresses cell wall synthesis, such as ethambutol, beta-lactams, isoniazid, and delamanid.

Brown-Eliot et al. published a practical overview of antimycobacterial agents and susceptibility breakpoints with minimum inhibitory concentration (MIC) for both slowly and rapidly growing nontuberculous mycobacteria [12]. Importantly, empirical treatment before NTM species identification should be avoided, as the recommended treatment regimen may differ substantially from one isolate to another. In general, the recommended drug combination for slowly growing NTM is a macrolide as the backbone, ethambutol, and a rifamycin. If the related infection is severe, aminoglycosides can be added [8,9,13]. The clinical efficacy of moxifloxacin and linezolid for slow growers is currently being investigated. For rapidly growing NTM, the drug therapy combination is primarily based on the in vitro results of DST. A DST usually includes macrolides, aminoglycosides, fluoroquinolones, amikacin, imipenem, tetracyclines, linezolid, trimethoprim–sulfamethoxazole, and tobramycin (for the *M. chelonae/M. immunogenum* complex only), but the clinical relevance of many DST results—with the exception of macrolides and aminoglycosides—is still a matter of debate [8,9,13,14]. Tigecycline may be tested, but there are insufficient data to establish MIC breakpoints. Therefore, for these three agents, if requested, a MIC without interpretation should be reported [12,15,16].

Other antibiotics that can be used for the treatment of NTM infections include clofazimine, bedaquiline, and tedizolid. Tedizolid is a new oxazolidinone antimicrobial that, like linezolid, inhibits protein synthesis by binding 23sRNA and has shown in vitro activity against some isolates of NTM, including some strains of the *M. abscessus* complex, *M. chelonae, M. mucogenicum*, the *M. fortuitum* group, *M. avium* complex, *M. kansasii,* and *M. marinum* [16]. Bedaquiline is a novel diarylquinolone antibiotic that offers a unique mechanism, i.e., inhibition of ATP synthase with rare cross-resistance to other agents. It has shown activity against a variety of mycobacteria, including multi-drug-resistant *M. tuberculosis*. Currently, there are no standardized procedures, breakpoints, or interpretive criteria for DST for clofazimine, tedizolid, or bedaquiline.

The rate of successful treatment is related to the species and type of infection (e.g., 30–50% in *M. abscessus*, 50–70% in *M. avium* complex, and 80–90% in *M. malmoense* and *M. kansasii* infections) (expert opinion). All patients with complex NTM infections receive multidrug regimens, frequently causing adverse reactions and/or drug–drug interactions. Treatment is always long, usually >12 months or even, as recommended in the NTM-PD guidelines. If there is no thorough source control, suboptimal or unfavorable outcomes are very likely. In conjunction with local surgical debridement and antimicrobial treatment, it is vital to evaluate the patient’s immunological status. Patients with immunodeficiency or comorbidities are found to be more susceptible to NTM disease, as discussed below.

## 6. Resistance Mechanisms in Mycobacteria and Drug Susceptibility Testing

When selecting a drug regimen for the treatment of NTM infection, it is important to know the possible resistance mechanisms—either intrinsically or acquired. NTM might be intrinsically resistant to certain antibiotics because of their structural nature, as they have an extremely hydrophobic and impermeable thick cell wall, while granuloma formation might also impair the influx of antibiotics [13]. There are four mechanisms proposed as the most probable ways of resistance in NTM infections: (a) decrease in antimicrobial influx (cell wall, affinity to bind to penicillin-binding protein (PBP); binding sites of peptidoglycan); (b) increase of antimicrobial efflux (efflux pumps, activity of Tet, Otr, Tap en LfrA); (c) induction of mutations (different genes); and (d) suppression of antimicrobial activity (interference with different enzymes) [13] (Figure 1).

Numerous studies have examined various facets of antibiotic resistance in highly pathogenic NTM isolates; however, many questions in this research area remain unanswered. One notable area of ongoing inquiry is the discordance between drug susceptibility testing (DST) and the actual phenotypic behavior of the organism. While DST provides valuable insights into the theoretical susceptibility of NTM to various antibiotics, it does not always accurately predict the organism’s in vivo response to treatment. For example, in *M. avium* complex isolates, there is no correlation between clinical response and in vitro MIC cutoff except for macrolides and amikacin [17]. Thus, DST prior to the initiation of treatment for *M. avium* should be limited to clarithromycin and amikacin. The second-line drugs, like moxifloxacin and linezolid, have uncertain clinical efficacy [12]. Similarly, MIC values for ethambutol and isoniazid do not correlate well with clinical response [12,18]. DST prior to the initiation of treatment for *M. kansasii* includes clarithromycin and rifampin susceptibility testing first and is expanded to other agents only if resistance is present. For the rapid growers, if there is amikacin resistance phenotypically detected, then sequencing for control of the 16sRNA mutation should be undertaken. Moreover, prolonged incubation is necessary for proper detection of an inducible *Erm* gene mutation and the evaluation of macrolide susceptibility [19]. A great number of the rapid growers are imipenem-susceptible, but this does not predict meropenem susceptibility.

DST is standard practice to predict macrolide susceptibility; however, in vitro DST and in vivo treatment responses do not always coincide for other antibiotics in mycobacteria. CLSI and ATS/IDSA have published criteria for performing DST [14]. The gold standard for determining DST is culture and growth via broth microdilution. In general, NTM DST should be performed only for clinically relevant isolates. For fast-growing mycobacteria, DST against a wider panel of antimicrobials is recommended. There are commercially available DST plates used by diagnostic reference laboratories, with distinct panels for rapid and slow growers. Normally, the plates include the following antibiotics: clarithromycin, rifabutin, ethambutol, isoniazid, moxifloxacin, rifampin, trimethoprim–sulfamethoxazole, amikacin, linezolid, ciprofloxacin, streptomycin, doxycycline, and ethionamide (for the slow growers); and cefoxitin, tigecycline, imipenem, cefepime, amoxicillin/clavulanic and tobramycin (for the fast growers). However, it is important to note that the purpose of this test is to help the clinician set up a combination of treatments, as there is no established MIC cut-off for susceptibility or resistance for most of these agents [16,17].

Whole genome sequencing (WGS) is required for accurate identification of the species/subspecies level of most NTM to better understand the basis of resistance. WGS can identify all single nucleotide polymorphisms (SNPs) associated with resistance as well as phylogenetic SNPs characteristic of individual NTM species [20]. In addition, WGS also contributes to the diagnosis of mixed NTM infections associated with two or more species [21]. Sequencing data can also be used in molecular epidemiology analysis to provide a detailed insight into the transmission dynamics of NTM [22]. In addition, WGS also allows the identification of NTM at the clone level, thus allowing the form (relapse/reinfection) of the ongoing disease to be determined [23]. In general, WGS results provided detailed information regarding the resistance, virulence, and persistence encoded on the chromosomes or plasmids of clinically important disease-causing NTM.

## 7. Host-Related Factors and Assessment of Immunity

NTM are typically considered to be of low pathogenicity. This perception arises from the fact that clinical diseases caused by NTM are relatively rare, despite their widespread presence in the environment. Consequently, it is essential to explore potential host susceptibility factors, whether hereditary or acquired, in NTM-infected patients.

The primary defense against mycobacteria involves a mechanical barrier composed of intact skin, mucosa, and localized antimicrobial responses. Any disruption in the structural integrity of these anatomical barriers, whether due to underlying comorbidities, surgical procedures, or trauma, increases the risk of secondary contamination and infection with environmental NTM. When these physical barriers are compromised, the host’s immune response serves as a secondary line of defense in the local management of NTM infection.

Effective immune-mediated clearance of mycobacterial infection relies on the coordinated interaction between the myeloid and lymphoid components. The host’s immune response to mycobacterial infection is primarily orchestrated by mononuclear phagocytes, which engulf and eradicate mycobacteria through a process involving progressive phagosome acidification and lysosomal fusion [24]. Upon internalization, infected macrophages generate interleukin IL-12, which subsequently binds to the IL-12 receptor (IL-12R), composed of two subunits, IL12-RB1 and IL-12RB2 [25]. The IL-12R is found on T cells and natural killer (NK) cells and initiates a downstream signaling cascade, facilitated by TYK2, leading to the production of the potent inflammatory cytokine interferon gamma (IFN-gamma) [25]. Macrophages express the heterodimeric IFN-gamma receptor, enabling downstream JAK-STAT signaling and instigating the further release of cytokines such as IL-12 and TNF-α [26]. This, in turn, amplifies the activation of macrophages, enhancing their intracellular killing of mycobacteria [26].

Inborn errors of immunity (IEIs) in the IL-12/IFN-gamma signaling axis predispose individuals to infection with weakly virulent mycobacteria. These diseases are referred to as mendelian susceptibility to mycobacterial disease (MSMD). Genetic mutations that impair IL-12/IFN-gamma host immunity against NTM include *IL12B*, *IL12RB1*, *ISG15*, *TYK2*, *IFNGR1*, *IFNGR2*, *JAK1*, *STAT1,* and *IRF8* genes [25,26,27,28]. In addition, protective immunity against mycobacteria is also mediated by the NFκB essential modulator (NEMO) pathway and the oxidative burst produced by macrophages, as shown by inherited *IKBG2* and *CYBB* mutations, respectively [29,30]. Finally, genetic deficiencies in several transcription factors responsible for regulating immune cell differentiation and function are associated with mycobacterial disease, including *GATA2*, *TBX21,* and *RORC* [31,32,33].

Individuals with MSMD are relatively uncommon and typically experience infections during their early years, spanning infancy, childhood, or young adulthood. While diagnostic evaluations may involve immunoassays that assess basal receptor expression or cytokine release upon stimulation, such testing is not consistently accessible [26]. Genetic testing remains the definitive gold standard for diagnosing these IEIs [26].

The emergence of late-onset NTM disease in patients typically indicates an acquired rather than a primary immunodeficiency. A striking example is the compromised CD4 T-cell-mediated immunity observed in HIV-infected patients, rendering them susceptible to disseminated NTM disease, particularly the M. avium complex [34].

Disseminated NTM disease has also been observed in other severely immunocompromised groups, including individuals with cancer and recipients of hematologic and solid organ transplants [35]. Furthermore, the widespread usage of immunosuppressive treatments in autoimmune and autoinflammatory disorders, encompassing targeted biological therapies and broad-spectrum immunosuppressants, has expanded the pool of patients at risk of NTM infection [36]. Agents associated with a theoretically high risk of NTM infection comprise the use of corticosteroids and monoclonal antibodies targeting TNF, IL-12/IL-23, CD3, CD52, and CTLA-4, as well as small molecules such as JAK inhibitors [36]. A comprehensive population-wide study conducted in Canada unveiled a significant increase in NTM infections, primarily of pulmonary origin, in patients receiving anti-TNFα therapy (including infliximab, adalimumab, and etanercept) for rheumatoid arthritis [37]. This study also identified other medications like leflunomide, hydroxychloroquine, and oral corticosteroids as contributors to an elevated risk of NTM infection [37].

Another distinct acquired immunodeficiency associated with susceptibility to NTM disease has been observed in patients who produce autoantibodies against IFN-gamma [38]. In the presence of these autoantibodies, the natural levels of IFN-gamma in response to mycobacterial infections are markedly diminished, making individuals susceptible to disseminated extrapulmonary mycobacterial infections [39]. This acquired impairment in the IFN-gamma pathway may help elucidate why certain patients are vulnerable to these intracellular pathogens, even in the absence of genetically inherited immunological deficiencies or evident acquired immunodeficiency.

Finally, individuals who appear ‘immunocompetent’ may still face an increased susceptibility to NTM disease. This susceptibility can be linked to host-related factors, including advanced age, malnutrition, and comorbidities like mitral valve prolapse, thoracic skeletal abnormalities, diabetes, arterial hypertension, and lung disease [35]. Whether this association primarily results from a higher prevalence of structural tissue diseases or also encompasses underlying immune dysfunction is a question that remains to be elucidated.

In this small case series, several patients are included with a diverse clinical picture as well as comorbidities. One can only speculate about the causal correlation between age, auto-IFN-gamma antibodies, and NK cell dysfunction in the process of immunosenescence as there are no specific laboratory data available. Furthermore, there are patients on specific immunomodulatory treatments (i.e., corticosteroids and posttransplant treatment). It is, therefore, important to think about diagnosing NTM infection in patients presenting with disseminated disease. On the other hand, it is reasonable to assess the risk factors of patients who present with severe or disseminated disease, including those with no prior diagnosis of immunodeficiency. This can influence the prognosis and may affect treatment decisions and duration.

## 8. Future Perspectives and Host-Directed Therapy (HDT)

NTM treatment with the current antimicrobial regimens is challenging, takes longer, and, in general, has a low cure rate because of antibiotic resistance and toxicity. Neutralizing pathogen-induced immune modulation by HDT is a promising therapy for current antibiotic treatments to combat intracellular mycobacterial infections, as they might also reduce morbidity by modulating inflammation and tissue destruction. The evidence available for host–pathogen interaction therapies result from TB studies. There are currently multiple active clinical trials evaluating the potential effect of HDT as adjunctive therapy to the standard treatment in *M. tuberculosis* infections (clinicaltrials.gov), such as using NSAIDs, COX-inhibitors (increase reactive oxygen species), everolimus (shown to be an inductor of autophagy), doxycycline (as a matrix metalloproteinase inhibitor), epigenetic modifiers, etc. [40,41]. Moreover, bedaquilline is shown to activate macrophages and increase their lysosomal function [42]. In a French study by Giraud-Gatineau A et al., with a combination of imaging and gene expression analysis, it was shown that exposure to bedaquiline increases phagosome–lysosome fusion of macrophages and their ability to autophagy with no effect on bacterial cells [42]. Therefore, bedaquiline can be regarded as a host-directed therapy for bacterial infections. Additionally, there is a recently published article revisiting the idea of using specifically mesenchymal stem cell infusion in patients with mycobacterial infection with a successful effect to treat MDR TB infection as a host-directed therapy [43]. The immunomodulatory effects of stem cells and their involvement in various infectious diseases result from their interactions with different immune cells in the body, such as T cells, B cells, macrophages, dendritic cells, and NK cells. In particular, in mycobacterial infections, the granuloma, a well-defined structure, is composed of these cells. There is little knowledge of the interactions of stem cells with different immune cells within the granuloma, which is a major obstacle to understanding their persistence. These types of stem cells may also be a potentially interesting therapeutic approach for different infections due to their potential to interact with the immune system [43].

For patients with anti-IFN gamma autoantibody and disseminated extrapulmonary NTM infections, administration of IFN-gamma, immune globulin, and plasmapheresis has been reported to be unsuccessful [44]. However, there is a case report from Japan where a patient had anti-IFN gamma autoantibody and was treated with Rituximab as an HDT, with observation of a reduction in autoantibody titer, improvement in IFN-gamma signaling, and clinical remission [45].

The limited number of NTM experimental models investigating host modulation and HDT emphasizes the need and urgency to understand NTM pathogenesis as well as identify potentially relevant host targets [43].

## 9. Conclusions

Extrapulmonary NTM infections were, in the past, mostly connected to HIV/AIDS. Currently, in view of the broader use of immunosuppressive treatments as well as an aging population, the prevalence of NTM infection will most likely continue to rise, not only in the lungs. There is an emerging need for clinicians to be aware of extrapulmonary NTM infections and the importance of timely diagnosing them and relying on different and complex steps of identification. DST and discussion with NTM experts are strongly advised to determine the best treatment combination, remembering that every patient needs a tailor-made treatment. To better manage these complicated infections, proper data are urgently needed on incidence, prevalence, and outcomes. This article can be regarded as a call to clinicians and researchers to consider starting a national registry of data on extrapulmonary NTM infection to help guide their treatment in the future.

## Figures and Tables

**Table 1:** Patient characteristics.

Patient	Age/Sex	Pathogen	Anatomic Site	Comorbidity	Surgery	Outcome
1	57y/M	*M. chimaera*	Thoracic vertebra	Mitral valve surgery	Yes	Favorable
2	70y/F	*M. xenopi*	Thoracic vertebra	Minimal change kidney disease and nephrotic syndrome Chronic corticosteroid therapy	Yes	Unfavorable
3	55y/M	*M. marinum*	Distal interphalangeal joint	Status after kidney transplant Treatment with tacrolimus, corticosteroids, and mycophenolate mofetil	Yes	Favorable
4	82y/F	*M. abscessus*	Lisfranc joint right foot	Degenerative arthrosis, Older age	Yes	Favorable
5	78y/M	*M. chelonae*	Medial malleoli left foot	RA negative polyarthritis and systemic sclerosis Chronic treatment with Corticosteroids	Yes, amputation	Unfavorable
6	32y/M	*M. fortuitum*	Left knee	No	Yes	Favorable

**Table 2:** The most used drugs with microbiologic activity against NTM.

Antibiotic	Mode of Activity	Mechanism of Action	Administration
Macroliden	bacteriostatic	Protein synthesis inhibition by	Oral/Intraveneus
		supression of peptide chain elongation	
		in ribosome	
Ethambutol	bacteriostatic	Cell wall synthesis inhibition	Oral
		Suppression of arabinogalactan	
		synthesis	
Rifamycin	bactericide	RNA polymerase inhibition	Oral
Quinolonen	bactericide	DNA gyrase inhibition	Oral
Aminoglycosiden	bactericide	Protein synthesis inhibition by	Intravenous
		suppression of translation process	
Carbapenem	bactericide	Peptidoglycan synthesis inhibition and	Intravenous
		low PBP activity	
Tetracyclines	bacteriostatic	Protein synthesis inhibition by	Oral/Intraveneus
		suppression of binding tRNA and mRNA	
		ribosome complex	
Sulfamethoxazole	bactericide	Folate synthesis pathway inhibition and	Oral
/Trimethoprim		nucleic acid synthesis	
Linezolid	bacteriostatic	Protein synthesis inhibition by binding	Oral
		23sRNA	
Clofazimine	bacteriostatic	Blocking the inracellular redox system	Oral
Thionamide	bactericide	Inhibit the mycobacterial synthesis of mycolic acid through a specific action against the inhA product enoyl-acyl carrier protein reductase;	Oral
Bedaquilline	bacteriostatic	mATP synthesis inhibition and lack of	Oral
		ATP production	

## Data Availability

The data presented in this study are available on request from the corresponding author.

## References

[1] Dahl V.N., Dahl V.N., Mølhave M., Mølhave M., Fløe A., Fløe A., van Ingen J., van Ingen J., Schön T., Schön T. (2022). Global trends of pulmonary infections with nontuberculous mycobacteria: A systematic review. Int. J. Infect. Dis..

[2] Matsumoto Y., Kinjo T., Motooka D., Nabeya D., Jung N., Uechi K., Horii T., Iida T., Fujita J., Nakamura S. (2019). Comprehensive subspecies identification of 175 nontuberculous mycobacteria species based on 7547 genomic profiles. Emerg. Microbes Infect..

[3] Tortoli E., Meehan C.J., Grottola A., Serpini G.F., Fabio A., Trovato A., Pecorari M., Cirillo D.M. (2019). Genome-based taxonomic revision detects a number of synonymous taxa in the genus Mycobacterium. Infect. Genet. Evol..

[4] Falkinham J.O. (2015). Environmental sources of nontuberculous Mycobacteria. Clin. Chest Med..

[5] CDC Website. https://www.cdc.gov/hai/organisms/ntm/clinicians.html#print.

[6] Piersimoni C., Scarparo C. (2009). Extrapulmonary infections associated with nontuberculous mycobacteria in immunocompetent persons. Emerg. Infect. Dis..

[7] Cruz-Aguilar M., Castillo-Rodal A.I., Arredondo-Hernández R., López-Vidal Y. (2021). Non-tuberculous mycobacteria immunopathogenesis: Closer than they appear. a prime of innate immunity trade-off and NTM ways into virulence. Scand. J. Immunol..

[8] Daley C.L., Iaccarino J.M., Lange C., Cambau E., Wallace R.J., Andrejak C., Böttger E.C., Brozek J., Griffith D.E., Guglielmetti L. (2020). Treatment of nontuberculous mycobacterial pulmonary disease: An official ATS/ERS/ESCMID/IDSA clinical practice guideline. Eur. Respir. J..

[9] Lange C., Böttger E.C., Cambau E., E Griffith D., Guglielmetti L., van Ingen J., Knight S.L., Santin M., E Stout J., Tortoli E. (2022). Consensus management recommendations for less common non-tuberculous mycobacterial pulmonary diseases. Lancet Infect. Dis..

[10] Quan T.P., Bawa Z., Foster D., Walker T., del Ojo Elias C., Rathod P., Iqbal Z., Bradley P., Mowbray J., MMM Informatics Group (2018). Evaluation of whole-genome sequencing for mycobacterial species identification and drug susceptibility testing in a clinical setting: A large-scale prospective assessment of performance against line probe assays and phenotyping. J. Clin. Microbiol..

[11] Lindeboom J.A., Kuijper E.J., van Coppenraet E.S.B., Lindeboom R., Prins J.M. (2007). Surgical excision versus antibiotic treatment for nontuberculous mycobacterial cervicofacial lymphadenitis in children: A multicenter, randomized, controlled trial. Clin. Infect. Dis..

[12] Brown-Elliott B.A., Woods G.L. (2019). Antimycobacterial susceptibility testing of nontuberculous mycobacteria. J. Clin. Microbiol..

[13] Tarashi S., Siadat S.D., Fateh A. (2022). Nontuberculous Mycobacterial Resistance to Antibiotics and Disinfectants: Challenges Still Ahead. BioMed Res. Int..

[14] Clinical and Laboratory Standards Institute (2018). Susceptibility Testing of Mycobacteria, Nocardia spp., and Other Aerobic Actinomycetes.

[15] Brown-Elliott B.A., Wallace R.J. (2012). Antimicrobial susceptibility testing, drug resistance mechanisms, and therapy of infections with nontuberculous mycobacteria. J. Clin. Microbiol. Clin. Microbiol. Rev..

[16] Brown-Elliott B.A., Wallace R.J. (2017). In vitro susceptibility testing of tedizolid against nontuberculous mycobacteria. J. Clin. Microbiol..

[17] Egelund E.F., Fennelly K.P., Peloquin C.A. (2015). Medications and monitoring in nontuberculous mycobacteria infections. Clin. Chest Med..

[18] Woods G.L., Brown-Elliott B.A., Conville P.S., Desmond E.P., Hall G.S., Lin G., Pfyffer G.E., Ridderhof J.C., Siddiqi S.H., Wallace R.J. (2011). Susceptibility Testing of Mycobacteria, Nocardiae, and Other Aerobic Actinomycetes.

[19] Nash K.A., Wallace R.J., Brown-Elliott B.A. (2009). A novel gene, erm(41), confers inducible macrolide resistance to clinical isolates of Mycobacterium abscessus but is absent from Mycobacterium chelonae. Antimicrob. Agents Chemother..

[20] Yoon J.-K., Kim T.S., Kim J.-I., Yim J.-J. (2020). Whole genome sequencing of Nontuberculous Mycobacterium (NTM) isolates from sputum specimens of co-habiting patients with NTM pulmonary disease and NTM isolates from their environment. BMC Genom..

[21] Hirabayashi R., Nakagawa A., Takegawa H., Tomii K. (2019). A case of pleural effusion caused by Mycobacterium fortuitum and Mycobacterium mageritense coinfection. BMC Infect. Dis..

[22] Walker T.M., Merker M., Knoblauch A.M., Helbling P., Schoch O.D., van der Werf M.J., Kranzer K., Fiebig L., Kröger S., Haas W. (2018). A cluster of multidrug-resistant Mycobacterium tuberculosis among patients arriving in Europe from the Horn of Africa: A molecular epidemiological study. Lancet Infect. Dis..

[23] Fukushima K., Kitada S., Matsumoto Y., Komukai S., Kuge T., Kawasaki T., Matsuki T., Motooka D., Tsujino K., Miki M. (2021). Serum GPL core antibody levels are associated with disease activity and treatment outcomes in Mycobacterium avium complex lung disease following first line antibiotic treatment. Respir. Med..

[24] Weiss G., Schaible U.E. (2015). Macrophage defense mechanisms against intracellular bacteria. Immunol. Rev..

[25] Rosenzweig S.D., Holland S.M. (2005). Defects in the interferon-γ and interleukin-12 pathways. Immunol. Rev..

[26] Wu U.-I., Holland S.M. (2015). Host susceptibility to non-tuberculous mycobacterial infections. Lancet Infect. Dis..

[27] Eletto D., Burns S.O., Angulo I., Plagnol V., Gilmour K.C., Henriquez F., Curtis J., Gaspar M., Nowak K., Daza-Cajigal V. (2016). Biallelic JAK1 mutations in immunodeficient patient with mycobacterial infection. Nat. Commun..

[28] Minegishi Y., Saito M., Morio T., Watanabe K., Agematsu K., Tsuchiya S., Takada H., Hara T., Kawamura N., Ariga T. (2006). Human tyrosine kinase 2 deficiency reveals its requisite roles in multiple cytokine signals involved in innate and acquired immunity. Immunity.

[29] Salt B.H., Niemela J.E., Pandey R., Hanson E.P., Deering R.P., Quinones R., Jain A., Orange J.S., Gelfand E.W. (2008). IKBKG (nuclear factor-κB essential modulator) mutation can be associated with opportunistic infection without impairing Toll-like receptor function. J. Allergy Clin. Immunol..

[30] Bustamante J., A Arias A., Vogt G., Picard C., Galicia L.B., Prando C., Grant A.V., Marchal C.C., Hubeau M., Chapgier A. (2011). Germline CYBB mutations that selectively affect macrophages in kindreds with X-linked predisposition to tuberculous mycobacterial disease. Nat. Immunol..

[31] Yang R., Mele F., Worley L., Langlais D., Rosain J., Benhsaien I., Elarabi H., Croft C.A., Doisne J.-M., Zhang P. (2020). Human T-bet Governs Innate and Innate-like Adaptive IFN-γ Immunity against Mycobacteria. Cell.

[32] Okada S., Markle J.G., Deenick E.K., Mele F., Averbuch D., Lagos M., Alzahrani M., Al-Muhsen S., Halwani R., Ma C.S. (2015). Impairment of immunity to Candida and Mycobacterium in humans with bi-allelic RORC mutations. Science.

[33] Vinh D.C., Patel S.Y., Uzel G., Anderson V.L., Freeman A.F., Olivier K.N., Spalding C., Hughes S., Pittaluga S., Raffeld M. (2010). Autosomal dominant and sporadic monocytopenia with susceptibility to mycobacteria, fungi, papillomaviruses, and myelodysplasia. Blood.

[34] Wetzstein N., Geil A., Kann G., Lehn A., Schüttfort G., Kessel J., Bingold T.M., Küpper-Tetzel C.P., Haberl A., Graf C. (2021). Disseminated disease due to non-tuberculous mycobacteria in HIV positive patients: A retrospective case control study. PLoS ONE.

[35] Henkle E., Winthrop K.L. (2015). Nontuberculous mycobacteria infections in immunosuppressed hosts. Clin. Chest Med..

[36] Lake M.A., Ambrose L.R., Lipman M.C.I., Lowe D.M. (2016). “Why me, why now?” Using clinical immunology and epidemiology to explain who gets nontuberculous mycobacterial infection. BMC Med..

[37] Brode S.K., Jamieson F.B., Ng R., A Campitelli M., Kwong J.C., Paterson J.M., Li P., Marchand-Austin A., Bombardier C., Marras T.K. (2015). Increased risk of mycobacterial infections associated with anti-rheumatic medications. Thorax.

[38] Kampmann B., Hemingway C., Stephens A., Davidson R., Goodsall A., Anderson S., Nicol M., Schölvinck E., Relman D., Waddell S. (2005). Acquired predisposition to mycobacterial disease due to autoantibodies to IFN-γ. J. Clin. Investig..

[39] Chen Y.-C., Weng S.-W., Ding J.-Y., Lee C.-H., Ku C.-L., Huang W.-C., You H.-L., Huang W.-T. (2022). Clinicopathological Manifestations and Immune Phenotypes in Adult-Onset Immunodeficiency with Anti-interferon-γ Autoantibodies. J. Clin. Immunol..

[40] Kilinc G., Saria A., Ottenhoff THM et Haks M.C. (2020). Host-directed therapy to combat mycobacterial infections. Immunol. Rev..

[41] Abe Y., Fukushima K., Hosono Y., Matsumoto Y., Motooka D., Ose N., Nakamura S., Kitada S., Kida H., Kumanogoh A. (2020). Host Immune Response and Novel Diagnostic Approach to NTM Infections. Int. J. Mol. Sci..

[42] Giraud-Gatineau A., Coya J.M., Maure A., Biton A., Thomson M., Bernard E.M., Marrec J., Gutierrez M.G., Larrouy-Maumus G., Brosch R. (2020). The antibiotic bedaquiline activates host macrophage innate immune resistance to bacterial infection. eLife.

[43] Devi A., Pahuja I., Singh S.P., Verma A., Bhattacharya D., Bhaskar A., Dwivedi V.P., Das G. (2023). Revisiting the role of mesenchymal stem cells in tuberculosis and other infectious diseases. Cell. Mol. Immunol..

[44] Browne S.K., Zaman R., Sampaio E.P., Jutivorakool K., Rosen L.B., Ding L., Pancholi M.J., Yang L.M., Priel D.L., Uzel G. (2012). Anti-CD20 (rituximab) therapy for anti–IFN-γ autoantibody–associated nontuberculous mycobacterial infection. Blood.

[45] Czaja C.A., Merkel P.A., Chan E.D., Lenz L.L., Wolf M.L., Alam R., Frankel S.K., Fischer A., Gogate S., Perez-Velez C.M. (2013). Rituximab as Successful Adjunct Treatment in a Patient With Disseminated Nontuberculous Mycobacterial Infection Due to Acquired Anti-Interferon- Autoantibody. Clin. Infect. Dis..

